# The UK’s Tobacco and Vapes Act puts the end of smoking within reach, but the job is not done

**DOI:** 10.1016/j.lanepe.2026.101781

**Published:** 2026-07-14

**Authors:** Sarah E. Jackson, Hazel Cheeseman, Jamie Brown

**Affiliations:** a Department of Behavioural Science and Health, University College London, London, UK; b Action on Smoking and Health, London, UK; c Behavioural Research UK, London, UK

The UK’s Tobacco and Vapes Act marks a defining moment in tobacco control. Its smokefree generation policy—prohibiting the sale of all tobacco products to anyone born on or after 1 January 2009—represents one of the most ambitious attempts globally to end the cycle of smoking uptake. By progressively raising the age of sale, the policy could substantially reduce smoking initiation among future cohorts and accelerate progress towards a smokefree society.

This approach builds on decades of successful tobacco control measures, which have driven smoking prevalence to historic lows.^1^ Yet smoking remains a leading cause of preventable illness and death, killing up to two thirds of lifelong users.^2^ The smokefree generation policy signals a shift from incremental reduction to a more explicit endgame strategy.

Modelling suggests restricting access to tobacco among younger generations could contribute to substantial long-term reductions in smoking prevalence.^3^^,^^4^ Evidence from raising the legal age of sale—e.g., from 16 to 18 in England and to 21 in the US—indicates that such measures can reduce smoking uptake among young people.^5^ By targeting initiation, the policy addresses smoking at its source.

However, the policy’s impact will depend critically on implementation. Retailer compliance and effective enforcement of age-of-sale restrictions will be essential,^6^ alongside behaviour change in the population permanently excluded from being sold tobacco, and clear communication with the public about the rationale for and nature of the policy. Arguments that the legislation could expand illicit tobacco markets (thus undermining the Act’s intentions) come largely from the tobacco industry.^7^ The Act introduces stronger powers to tackle illicit tobacco sales, including the potential for a retail licensing scheme. Continued investment in enforcement will be important.

Equity considerations are central. Smoking prevalence is concentrated in more disadvantaged groups,^1^ contributing substantially to health inequalities. While a policy focused on future generations is important, it does not directly address the needs of more than 5 million adults in the UK who currently smoke.^1^ This population—which may be larger than official estimates suggest^8^^,^^9^—will continue to experience smoking-related harm without sustained and targeted action. The government has committed to maintaining funding to support smokers to quit and this continued investment, particularly for high-prevalence groups, will be needed to avoid the risk that inequalities could widen further. Ensuring equitable access to effective stop-smoking support much remain a priority.

How the policy is understood and communicated will also shape its impact. There is a risk that such a landmark measure is seen as marking the end of the tobacco problem. In reality, success depends on maintaining momentum across tobacco control and cessation support more broadly. Without this, progress in reducing prevalence could stall and inequalities persist.

At the same time, the legislation offers a rare opportunity to generate renewed national attention on smoking and its harms, creating a moment to encourage quitting at scale, as seen following the introduction of smokefree legislation in 2007.^10^ Framing the policy as a major step forward, rather than a final destination, will be important in maintaining political will, sustaining investment in cessation support, and maximising public health impact.

Passage of this legislation represents both an achievement and an opportunity. It creates a foundation for further progress but does not remove the need for continued effort. Priorities include robust implementation and enforcement; sustained investment in evidence-based cessation services; targeted action for groups with persistently high smoking rates; evaluation of impacts and unintended consequences; and continued adaptation of regulatory approaches in response to industry behaviour and emerging evidence. The latter could include measures to reduce industry influence and ensure sustainable funding for tobacco control, such as a ‘polluter pays’ levy on tobacco manufacturers.

The UK will become only the second country to implement a generational ban on tobacco sales, after the Maldives implemented similar legislation in November 2025. As an early adopter with strong surveillance and evaluation infrastructure, the UK is well placed to generate high-quality evidence to inform policy elsewhere in the world. If successful, the smokefree generation policy could provide a model for moving beyond traditional tobacco control towards endgame strategies.

The Tobacco and Vapes Act is a landmark step towards a smokefree future. Realising its full potential will require sustained commitment, careful implementation, monitoring and evaluation, and continued focus on those most affected by smoking-related harm. The end of smoking is now more imaginable than ever, but it is not yet guaranteed.

## Contributors

Jackson: conceptualisation, writing—original draft, writing—review and editing. Cheeseman: writing—review and editing. Brown: conceptualisation, funding acquisition, writing—review and editing.

## AI disclosure

ChatGPT was used to assist with refining language.

## Declaration of interests

SEJ receives salary support from Cancer Research UK (PRCRPG-Nov21∖100002) and holds a leadership role (Past President) for the Society for Research on Nicotine and Tobacco Europe. HC is Chief Executive of Action on Smoking and Health, which has received funding from Cancer Research UK and the British Heart Foundation. JB has received programme grant funding from Cancer Research UK. All authors declare no financial links with tobacco companies, e-cigarette manufacturers, or their representatives.
